# The Healthy Taiwanese Eating Approach is inversely associated with all-cause and cause-specific mortality: A prospective study on the Nutrition and Health Survey in Taiwan, 1993-1996

**DOI:** 10.1371/journal.pone.0251189

**Published:** 2021-05-06

**Authors:** Shao-Yuan Chuang, Hsing-Yi Chang, Hsin-Ling Fang, Shu-Chen Lee, Yueh-Ying Hsu, Wen-Ting Yeh, Wen-Ling Liu, Wen-Harn Pan

**Affiliations:** 1 Institute of Population Health Science, National Health Research Institutes, Miaoli, Taiwan, R.O.C; 2 Institute of Public Health, National Yang Ming University, Taipei, Taiwan, R.O.C; 3 Institute of Biomedical Sciences, Academic Sinica, Taipei, Taiwan, R.O.C; University of Hong Kong, HONG KONG

## Abstract

**Background:**

Few longitudinal studies have investigated the association between foods/dietary pattern and mortality risk in the Asian population. We investigated the prospective association between foods/dietary pattern and risk of death among ethnic Chinese adults in Taiwan.

**Methods:**

The study population included 2475 young and middle-aged adults (aged 18–65 years at baseline) who completed the questionnaires and physical examinations in the Nutrition and Health Survey in Taiwan from 1993 to 1996. A food frequency questionnaire was administered to assess food consumption habits in a face-to-face interview. With survey data linked to the Taiwanese Death Registry, Cox proportional hazard model was used to identify the foods associated with all-cause mortality(followed until 2012), which were then tallied to calculate a dietary pattern score called Taiwanese Eating Approach(TEA) score. The TEA scores were then associated with various kinds of mortality outcomes. In addition, data from 431 elders (aged≥65 yrs) with 288 death endpoints were used to conduct a sensitivity analysis.

**Results:**

A total of 385(15.6%) participants died (111 cardiovascular related deaths and 122 cancer related deaths) during the 17.8-year follow-up period(41274 person-years). Twelve foods (9 inverse [vegetables/fish/milk/tea](+1) and 3 positive[fatty meats/fermented vegetables/sweet drinks](-1)) were significantly associated with all-cause mortality risk. All adults were grouped by their cumulative food score into three diet groups: poor diet(29.3% of all subjects), average diet(44.0%), and healthy diet(26.70%). The better the diet, the lower the total, cardiovascular, and other cause mortality outcomes (trend-*p* < .001). The hazard ratio for the healthy diet was 0.64 (95% confidence interval:0.47–0.87) for total mortality, and 0.52(0.28–0.95) for cardiovascular death, compared with the poor diet in the multivariable models. This phenomenon was also seen in older adults for all-cause, cancer, and other cause mortalities.

**Conclusion:**

Consuming a healthy Taiwanese Eating Approach (TEA) diet is negatively associated with all-cause, cardiovascular, and other-cause mortalities in Taiwan.

## Introduction

Cardiovascular diseases (CVDs) are the most common cause of death globally [1], and most CVDs can be prevented by changing behavioral risk factors such as tobacco use, unhealthy diet, and physical inactivity. Furthermore, according to epidemiological measures [2], dietary factors contribute to 30–60% of the excess risk of developing cancers. A nutritious diet and routine exercise are well documented to exhibit beneficial effects on longevity [3]. However, foods and dietary habits vary by culture and geographical areas [4], and the relationships between mortality and regional diets have not been thoroughly examined.

Although viewpoints are mixed concerning what constitutes a nutritious diet for maintaining or achieving health [5, 6], investigators are still devoted to identifying and reconfirming foods or dietary patterns that are associated with health or disease, such as the Mediterranean diet or the Dietary Approach to Stop Hypertension (DASH). Investigating associations between diet and mortality is a valid endeavor The Mediterranean diet has been demonstrated to have a significant beneficial effect on risk reduction of cancer, cardiovascular mortality, and geriatric syndrome [7*–*9]. In addition, the DASH diet has been shown to have significant benefits on reducing both blood pressure and blood lipid levels [10] and to reduce the risk of stroke [11], osteoporosis [12], and geriatric syndrome [13]. These two diets are popular in western countries, but people in Asian countries consume diets with very different components under distinctive dietary cultures [14, 15]. For example, Asian populations consume less beef, dairy products, olive oil, butter, and coffee, but more vegetables, soy-products, rice, and tea. Therefore, food choices for achieving good health in Asia may differ from those espoused in the Mediterranean or European regions.

Studies investigating the association between diet and longevity in Asian populations have been rare and inconclusive, especially concerning which foods or dietary patterns lead to better health outcomes [16*–*18]. Therefore, we carried out a prospective study in young and middle-aged adults to investigate the relationship between foods/dietary patterns and mortality with respect to all cause, CVD, cancer, and other causes in Taiwan. We validated the findings using a subgroup analysis of older adults.

## Methods

### Study population

Data for the study population were derived from the Nutrition and Health Survey in Taiwan (NAHSIT) between 1993 and 1996. The sampling methods of this survey have been described [19]. In brief, 359 township or city districts in Taiwan were classified into 7 strata according to their dietary pattern, geographical location, and degree of urbanization. A total of 9978 individuals, ages 4 and above, were interviewed with a total response rate of 74%. Among those interviewed, 64.4% (6245 individuals) participated and completed the physical examination and 5304 subjects completed the food frequency questionnaire (FFQ) (S1 Fig). We excluded children and adolescents (age ≤ 18 years) to focus on adults. We used data from an elder population (≥65 years) to conduct the sensitivity analysis. Due to fewer food items were included in the FFQ for elders compared to that for young adults and some contents of the FFQ were different between adults and elderly populations.

As such, the final study population included 2475 individuals between ages 18 and 65 years for identifying and constructing the dietary scores/patterns, which were then associated with mortality. The data from 431 elders aged ≥ 65 years were used for a sensitivity analysis to validate the dietary score-mortality associations. A research ethics committee nor an Institutional Review Board were established in Taiwan from 1993–1996, so we did not require written informed consent. All participants in this study were verbally informed of the purposes of the Nutrition and Health Survey in Taiwan from 1993–1996 and verbal consent was obtained from every participant at interview. All procedures in this study were performed in accordance with relevant regulations, which were approved by the Research Ethics Committee of the National Health Research Institutes (EC1030801-E).

### Food frequency questionnaire

Data for the frequency of food consumption in the month prior to the examination were collected by well-trained interviewers using a simple, 28-item food frequency questionnaire (FFQ), which was validated [20]. Spearman’s rank correlation coefficients between frequencies of food groups obtained from the FFQ and from dietary recall ranged from 0.132 to 0.678 for males; 0.052 to 0.759 for females [20]. Food items assessed in the questionnaire included freshwater fish, poultry (chicken, duck), lean meat (pork), high-fat meat (marbled red meats with skins or high fat beef), animal liver, other organ meats, eggs, deep-ocean fish (with rich OMGEA-3), shellfish, other seafood [shrimp or crab], soy products (tofu and its products), soy milk, whole milk, skim milk, vegetables as a whole, carrots, melons, mushrooms, bamboo shoots, beans, dark-colored vegetables, seaweed, pickled/fermented vegetables, fruits as a whole, 100% fruit juice, soft drinks, tea, coffee and fermented foods. The frequency of consumption per week was calculated for all items and used for statistical analysis. The interviews to assess food frequency were conducted each month using an equal distribution to avoid a seasonal effect.

### Definition of variables

Education, which was determined by years in school, served as a surrogate of socio-economic status. Smoking was classified as none, former, and current smoking. Alcohol drinking habits were classified into none, former, current drinking and “refused to answer”. For physical activity, we used a structured questionnaire to assess frequency (times per week) and kinds (12 kinds of sports and open questions) of sports. We summed the frequency of all sports for each individual to evaluate physical activity level per week. Regular physical activity was defined as more than two physical activities per week. Body mass index (BMI) was calculated as bodyweight (kg) divided by height squared (m^2^). Obesity status was classified as BMI<18.5, 18.5 to 21.9, 22 to 23.9, 24 to 26.9, 27 to 29.9, and ≥30 kg/m2. We used bodyweight as a surrogate of total energy take [21]. Blood pressure was measured three consecutive times, and the average of the latter two measurements (or the closest two of the latter three on rare occasions) was used for analysis. To help maintain the size of the study population, missing values were imputed before statistical analysis (S2 Table in S1 File).

### Cohort follow-up and mortality ascertainment

After completing the baseline surveys, the dates and causes of death were collected by linking our database with data from the National Death Registry through a unique personal identification number which is given to every Taiwanese at birth. Participants who were not present in the National Death Registry at the end of the follow-up period (31 December 2012) were considered survivors. The National Death Registry database registers valid death information based on the certified death certificates for every Taiwanese. The death certificates were coded according to the International Classification of Disease, Ninth Revision (ICD-9) and Tenth Revision (ICD-10) and the primary death codes were used for this study. The cardiovascular disease codes for CVD in ICD-9 and ICD-10 are 390–459 and I00–I99, respectively, and the codes for cancer in ICD-9 are 140–239 and those for ICD-10 are C00–C99 or D00–D49.

### Statistical methods

We performed survival curves to establish the Taiwanese Eating Approach (TEA) scores based on data from the FFQ and national death registry in this prospective design. First, we used the Cox proportional hazard model to identify the foods that were significantly associated with all-cause death risk among individuals aged 18 to 65 years in the multivariable models with adjustment for age, sex, smoking, physical activity, and education. The inclusion criteria of candidate foods for further analysis were (1) food frequency [times/week] or dichotomous food consumption status (low/high consumption) with a p-value less than 0.1 in the multivariable model with adjustment for age, sex, smoking, physical activity, and education, and (2) no U-shaped association between food and mortality. In addition, the food with a very low consumption frequency in the population at the time was excluded (e.g., pumpkin).

We identified 9 healthy foods that were inversely and significantly associated with total mortality and 3 risky foods that were positively and significantly associated with total mortality. Individuals who consumed a healthy food more than once per week were given a score of 1 (positive one); on the contrary, those who consumed a risky food more than once per week were given a score of -1 (negative one). We generated a total food score by summing individual food scores and named this score the Taiwanese Eating Approach (TEA) score. Since this study was the first nationally representative study of food intake frequency, no prior eating approach has been named for Taiwan. We classified TEA scores into three diet groups, poor diet (TEA: -2~+2), average diet (TEA: 3~4) and healthy diet (TEA: ≥5).

After we established the TEA scores, we further estimated the association between TEA score and all-cause, cardiovascular, cancer and other-cause mortality using the Cox proportional hazard models. We used the Kaplan–Meier method to estimate the survival curves and used the log-rank test to examine the homogeneity of the survival curves. We then analyzed the characteristics and the frequency of food intake among the three groups. In addition to a crude model, we established four other models to assess the influence of potential confounding factors on the association between TEA score and CVD/cancer/other cause/all-cause mortality. The five models were: crude model; model 1: adjusted for age and sex; model 2 further adjusted for physical activity, smoking, drinking and education; model 3: adjusted for age, sex, exercise, smoking, drinking, education, obesity, and number of self-reported diseases; and model 4: further adjusted for metabolic disorders and chewing betel nuts (which was positively associated with total mortality). There were 17 adults with a history of diagnosed cancer. We further established a multivariable model among the adults without a cancer history (model 5). All models controlled for bodyweight as the surrogate of total energy intake. All statistical procedures were performed using SAS 9.4.

## Results

The 2475 adults between ages 18 and 65 years who underwent the physical examination for the NAHSIT were included in this study to investigate the association between diet and mortality. A total of 385 adults died (cumulative incidence rate = 15.56%) during the follow-up period (median follow-up: 17.78 years). Of these deaths, cardiovascular mortality accounted for 28.8% (n = 111) and cancer mortality for 31.7% (n = 122). Men had greater mortality risk than women for all-cause death (13.0 vs. 6.2, per 1000 person-years, PYs; p < 0.01), cardiovascular death (3.4 vs. 2.1, per 1000 PYs; p < 0.01), and cancer death (4.2 vs. 1.9, per 1000 PYs; p < 0.01).

Twelve foods (9 inversely and 3 positively) were significantly associated with the risk of all-cause mortality in the model with adjustment for age, sex, smoking, physical activity, and education (model 2). The inversely associated foods included sea fish with rich omega-3, freshwater fish, other seafood (shrimp or crabs), vegetables, and specific vegetable items such as seaweed and mushrooms, fruits, milk (whole or skim milk), and tea. Positively associated foods included fatty meats, fermented vegetables, and sweet beverages. We generated a TEA score for each participant based on the consumption status of these 12 foods. A single healthy food (consumed more than once a week) was assigned +1 point and a single risky food was assigned -1 point. TEA scores for all participants ranged from -2 to 7 points. We classified all participants into three diet groups based of their TEA score: -2~+2 points (group 1; the poor diet group, n = 726, 29.3%), 3 and 4 points (group 2; the average diet group, n = 1088, 44.0%), and 5–11 points (group 3; the healthy diet group, n = 661, 26.7%).(Table 1) The adults in the healthy diet group were younger, had lower BMI, waist circumference, blood pressure, uric acid, and triglycerides, but higher physical activity (Table 2).

**Table 1:** Characteristics of the participants from NAHSIT 1993–1996 by dietary quality score: On physical examination, laboratory test, and comorbidity parameters. The comorbidity cutoff point was defined as follows. Overweight/obesity: BMI ≥24 kg/m^2^; hypertension: systolic blood pressure ≥ 140 mmHg or diastolic blood pressure ≥ 90 mmHg or using medications; hypertriglyceridemia: triglycerides ≥200 mg/dL; hypercholesterolemia: total cholesterol ≥240 mg/dL; diabetes: glucose ≥126 mg/dL or taking anti-glucose medication; High LDL: ≥ 130 mg/dL. Low HDL: HDL<40/50 mg/dL.

	Total	Poor (N = 726) (TEA:-2~+2)	Average (N = 1088) (TEA:3~4)	Healthy(N = 661) (TEA≥5)	P-for trend*
	mean±std	mean±std	mean±std	mean±std	
Age, yrs	43.5±13.4	45.0±13.7	43.7±13.4	41.5±12.8	< .001
Sex (male %)	46.6%	52.6%	54.5%	52.7%	0.968
Height (cm)	160.5±8.2	159.3±8.0	160.6±8.4	161.8±7.9	< .001
Weight (kg)	61.5±10.9	61.7±10.6	61.6±11.2	61.0±10.6	0.185
BMI (kg/m^2^)	23.9±3.9	24.3±3.9	23.9±3.9	23.3±3.5	< .001
Waist circumference (cm)	77.7±10.4	79.1±10.5	77.8±10.4	76.0±10.0	< .001
Blood pressure					
Systolic (mmHg)	123.6±19.0	125.7±20.3	123.9±18.7	120.8±17.9	< .001
Diastolic (mmHg)	80.0±13.1	81.3±13.3	80.2±13.0	78.3±12.9	< .001
Current smoking (%)	24.3%	27.6%	23.3%	22.5%	0.028
Current drinking (%)	16.8%	21.0%	15.4%	14.5%	0.001
Physical activity (%)	58.1%	50.8%	59.5%	64.0%	< .001
Clinical chemistry					
Glucose (mg/dL)	87.9±25.7	88.5±26.6	88.3±25.9	86.7±24.3	0.225
Total cholesterol (mg/dL)	193.9±41.3	192.6±39.8	196.1±42.6	191.6±40.6	0.721
Triglyceride (mg/dL)	132.8±137.8	141.2±136.3	136.4±151.3	117.8±113.5	0.002
Uric acid (mg/dL)	6.2±1.8	6.3±1.9	6.2±1.8	6.0±1.7	0.007
HDL-C (mg/dL)	57.2±19.9	57.0±21.3	56.9±18.8	58.0±20.0	0.348
LDL-C (mg/dL)	111.7±37.6	109.3±37.3	113.8±37.6	110.9±37.8	0.415
**Comorbidity**					
Overweight/obesity (%)	45.0%	50.6%	44.8%	39.5%	< .001
Hypertension (%)	28.8%	33.1%	28.7%	24.4%	0.003
Diabetes (%)	5.6%	9.06%	5.70%	4.99%	0.391
Hypercholesterolemia (%)	11.3%	10.2%	11.9%	11.5%	0.428
Low HDL-C (%)	27.1%	26.3%	27.7%	26.9%	0.781
High LDL-C (%)	33.6%	34.0%	33.0%	34.0%	0.992
Hypertriglyceridemia (%)	13.3%	14.9%	13.3%	11.4%	0.053
Hyperuricemia (%)	36.4%	37.9%	37.4%	33.1%	0.071

*: The trend associations of age and comorbidities for diet groups based on the Taiwanese Eating Approach (TEA) score was evaluated by general linear regression with ordinal TEA as an independent variable.

**Table 2:** Individual characteristics by dietary quality score, food items and intake frequency by foods score group.

	Total	Poor (N = 726) (TEA:-2~+2)	Average (N = 1088) (TEA:3~4)	Healthy (N = 661) (TEA≥5)	
	Mean ± std	Mean ± std	Mean ± std	Mean ± std	p-value
TEA food consumption (times/week)					
**Healthy Foods**					
Sea Fish with rich OMEGA-3	0.96±1.71	0.40±0.9	0.87±1.6	1.73±2.2	< .001
Freshwater fish	4.59±6.46	3.23±8.4	4.74±4.8	5.86±6.1	< .001
Other fish (shrimp or crabs)	0.73±1.37	0.31±0.5	0.67±1.4	1.27±1.8	< .001
Seaweed	0.66±1.12	0.33±0.8	0.56±1.1	1.17±1.2	< .001
Mushrooms	1.02±1.69	0.49±0.9	1.02±1.9	1.60±1.8	< .001
Vegetables	18.06±12.21	16.81±14.6	18.39±11.7	18.88±9.8	0.001
Fruits	6.35±5.93	4.34±4.9	6.53±5.6	8.26±6.8	< .001
Milk	2.57±4.09	1.13±3.0	2.64±4.2	4.04±4.3	< .001
Tea	5.58±15.39	3.47±9.8	5.46±11.8	8.10±23.3	< .001
**Risky Foods**					
Fatty meats	2.17±3.44	2.81±4.0	2.03±3.3	1.68±2.9	< .001
Fermented vegetables	1.18±3.61	1.49±2.8	1.20±4.8	0.82±1.6	< .001
Sweetened drinks	3.30±6.16	3.76±6.3	3.22±6.5	2.92±5.3	0.011

The trend associations of food consumption for the three Taiwanese Eating Approach (TEA) score groups were evaluated by general linear regression with ordinal TEA as an independent variable.

**Fig 1:**
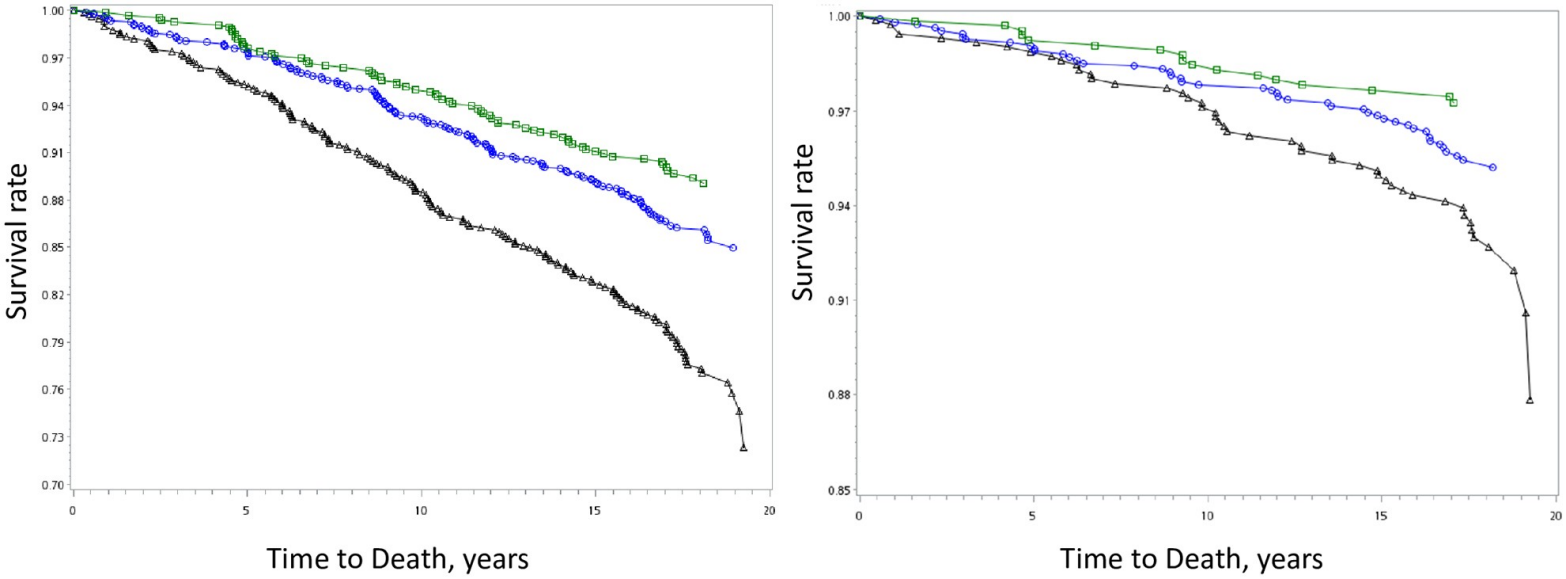
Survival Curves of The Healthy Taiwanese Eating Approach for (a) total and (b) cardiovascular mortality. A significant difference existed among three groups in total and cardiovascular mortality (both p-value less than 0.05). Taiwanese Eating Approach were classified into three groups, group 1 (black triangle, poor diet), group 2 (blue circle, average diet), and group 3 (green square, healthy diet).

Group 1 had the highest mortality from all causes, CVD, cancer, and other causes, and there was an association between TEA score and all-cause mortality (Fig 1). The mortality rates for groups 1, 2, and 3 were 13.9, 8.3, and 6.1 per 1000 PYs, respectively (Table 3; p-value for trend < 0.05). Compared with group 1, groups 2 and 3 corresponded to a 33% (hazard ratio: 0.67; 95% confidence intervals: 0.54~0.84) and a 46% (0.54; 0.41~0.72) low risk for all-cause mortality, respectively; a 28% (0.72; 0.48–1.07) and a 52% (0.48; 0.27~0.84) low risk for cardiovascular mortality, respectively; and a 15% (0.85; 0.57–1.28) and a 20% (0.80; 0.49–1.30) low risk for cancer mortality, respectively (Model 1 in Table 3).

**Table 3:** The association between the Taiwanese Eating Approach score and mortality (all causes, cancer, and cardiovascular mortality) among young adults (n = 2475). Model 1: adjusted for age and sex. Model 2: adjusted for age, sex, exercise, smoking, drinking, and education. Model 3: adjusted for age, sex, exercise, smoking, drinking, education, obesity, and number of self-reported diseases. Model 4: adjusted for age, sex, exercise, smoking, drinking, education, obesity, number of self-reported diseases, systolic BP, diastolic BP, triglycerides, HDL-C, LDL-C, sugar intake (times/week), and belt-nuts (times/week). Model 5: all independent variables included in model 4 among those without a history of cancer at baseline (n = 2458).

Diet group	Hazard ratio for Per units	Taiwanese Eating Approach			p-value for trend
		Poor (N = 726) (TEA:-2~+2)	Average (N = 1088) (TEA:3~4)	Healthy (N = 661) (TEA≥5)	
Mortality*					
All-cause	0.81 (0.76–0.85)	13.9	8.3	6.1	< .001†
CVD death	0.81(0.73–0.91)	4.1	2.6	1.5	< .001†
Cancer death	0.84 (0.76–0.93)	3.8	2.8	2.3	0.031
Other-cause	0.77 (0.70–0.85)	6.0	3.0	2.3	< .001†
Model 1					
All-cause	0.85 (0.80–0.90)	1.0 (REF)	0.67 (0.54–0.84)	0.54 (0.41–0.72)	< .001†
CVD death	0.87 (0.78–0.97)	1.0 (REF)	0.72 (0.48–1.07)	0.48 (0.27–0.84)	0.007†
Cancer death	0.90 (0.81–0.998)	1.0 (REF)	0.85 (0.57–1.28)	0.80 (0.49–1.30)	0.341
Other-cause	0.79 (0.72–0.87)	1.0 (REF)	0.54 (0.38–0.77)	0.43 (0.28–0.68)	< .001†
Model 2					
All-cause	0.87 (0.81–0.92)	1.0 (REF)	0.70 (0.56–0.87)	0.60 (0.45–0.81)	< .001†
CVD death	0.88 (0.78–0.98)	1.0 (REF)	0.73 (0.49–1.10)	0.51 (0.29–0.89)	0.014
Cancer death	0.90 (0.81–1.01)	1.0 (REF)	0.86 (0.57–1.29)	0.84 (0.51–1.38)	0.440
Other-cause	0.83 (0.75–0.91)	1.0 (REF)	0.58 (0.41–0.83)	0.53 (0.34–0.84)	0.002†
Model 3					
All-cause	0.87 (0.82–0.92)	1.0 (REF)	0.67 (0.54–0.84)	0.60 (0.45–0.80)	< .001†
CVD death	0.88 (0.79–0.99)	1.0 (REF)	0.68 (0.45–1.03)	0.50 (0.28–0.88)	0.009†
Cancer death	0.90 (0.81–1.01)	1.0 (REF)	0.85 (0.56–1.27)	0.83 (0.51–1.37)	0.428
Other-cause	0.83 (0.75–0.92)	1.0 (REF)	0.57 (0.40–0.81)	0.53 (0.34–0.84)	0.002†
Model 4					
All-cause	0.88 (0.82–0.94)	1.0 (REF)	0.67 (0.53–0.86)	0.62 (0.46–0.85)	< .001†
CVD death	0.88 (0.78–0.99)	1.0 (REF)	0.68 (0.44–1.06)	0.48 (0.26–0.88)	0.012†
Cancer death	0.92 (0.82–1.03)	1.0 (REF)	0.82 (0.52–1.28)	0.90 (0.53–1.52)	0.616
Other-cause	0.85 (0.76–0.94)	1.0 (REF)	0.57 (0.39–0.84)	0.56 (0.34–0.90)	0.005
Model 5					
All-cause	0.88 (0.82–0.94)	1.0 (REF)	0.69 (0.54~0.89)	0.64 (0.47~0.87)	< .001†
CVD death	0.88 (0.78~0.998)	1.0 (REF)	0.72 (0.46~1.13)	0.52 (0.28~0.95)	0.046
Cancer death	0.92 (0.82~1.04)	1.0 (REF)	0.87 (0.55~1.37)	0.92 (0.54~1.57)	0.178
Other-cause	0.85 (0.76~0.94)	1.0 (REF)	0.57 (0.39~0.84)	0.56 (0.34~0.91)	0.002†

*: Mortality, (1/1000 PY)

^†^: significance criterion (p-value = 0.0125 = 0.052/4) for multiple comparisons.

The trend associations of food consumption for the three Taiwanese Eating Approach (TEA) score groups were evaluated by general linear regression with ordinal TEA as an independent variable.

Those adults with a healthy diet (group 3) exhibited significantly lower risk for all-cause mortality, cardiovascular mortality and death from other causes compared to individuals with a poor diet (group 1) in the multivariable model adjusted for age, sex, exercise, smoking, drinking, and education (model 2). The significant reduction in mortality risk among those with the average diet and the healthy diet did not change in the multivariable models after further adjusting for potential confounding factors and specific risky foods (chewing betel nuts) (Model 4 in Table 3).

We further conducted a sub-group analysis to investigate the association between TEA score and mortality in an elderly population. Due to five elders having a physician-diagnosed cancer history at baseline, a total of 426 were for included for further analysis. Among the 426 elders aged >65 years, the follow-up period was 5088 person-years and all-cause, cardiovascular and cancer mortality were 56.6 (n = 288), 19.7 (n = 100), 11.0 (n = 56), per 1000 person-years respectively. Since the FFQ questionnaire used for elder adults included fewer items than the FFQ used with young/middle age adults, only 7 of the 12 mortality associated foods were covered in the elderly population. The TEA score among the elderly population included freshwater fish, fatty meat, milk, vegetables, fruits, tea, and fermented foods. The food score ranged from -1 to 5 and the average (standard deviation) was 2.79 (1.22). In the multivariable model, the hazard ratio of the TEA score was 0.84 (0.75–0.94, p = 0.0026) for all-cause mortality, 1.05 (0.85–1.28, p = 0.71) for cardiovascular mortality, 0.58 (0.44~0.75, p<0.001) for cancer mortality, and 0.85 (0.72~1.00, p = 0.05) for other-causes of death (S1 Table in S1 File).

## Discussion

### Main findings

Twelve mortality predictive foods were identified. The healthier foods includedomega-3 rich sea fish, freshwater fish, other seafood (shrimp or crab), vegetables as a whole along with specific vegetables such as seaweed and mushroom, fruits, milk, and tea, while the riskier foods included fatty red meats, fermented vegetables, and sweet drinks. The consumption frequency of these 12 foods were used to establish an individual TEA score, which was significantly associated with all-cause mortality and mortality from CVD and other causes dependent on the age groups studied over an 18-year follow-up period in a Chinese population living in Taiwan.

Moreover, this association was independent of potential confounding factors, such as total energy intake (bodyweight as a surrogate), socioeconomic status (education as a surrogate), risky behaviors (smoking, alcohol drinking and physical inactivity), and risky lifestyle (chewing betel nuts). This finding indicates that diet may have a significant and independent association with mortality.

### Studies in the Asian population

Few studies have investigated the association between dietary patterns in Asian populations, especially in ethnic Chinese, and mortality. The Singapore Chinese Health Study identified two major dietary patterns by principal component analysis: one was a vegetable-, fruit- and soy-rich (VFS) pattern, and the other was a dim sum- and meat-rich pattern [22]. Results of that study revealed that the VFS pattern was inversely associated with all-cause mortality and cause-specific mortality (cancer, CVD, and respiratory disease) during the follow-up period. The Japan Collaborative Cohort study revealed that ’vegetable’ and ’dairy product’ patterns were associated with lower mortality from CVD [23]. Our results are consistent with these previous results. We found that weekly consumption of vegetables, fruits, and dairy products was significantly associated with reduced all-cause mortality. We further identified other specific foods that were inversely associated with cardiovascular mortality, as summarized below.

### Vegetable/fruit intake and mortality

A vegetable-rich diet has been inversely associated with CVD [24, 25], cancer [26], and total mortality [27]. Our current results revealed that the consumption of seaweed and mushrooms were negatively associated with all-cause mortality. The negative association between seaweed and metabolic disorders and cancers has been reported in animal and human population studies. Seaweed supplements were found to normalize the levels of glucose, cholesterol, triglycerides, and systolic blood pressure in rats fed a high-carbohydrate, high-fat diet [28]. Furthermore, a randomized, double-blinded, placebo-controlled human trial revealed that seaweed intake of more than 4g per day for 1 month resulted in a significant decrease in systolic blood pressure and waist circumference [29]. The rich soluble fiber in seaweed may contribute to protection against cardiometabolic disorders [28]. However, because seaweed is rich in iodine, which might be a suspected risk factor for thyroid cancer, it remains controversial whether seaweed supplementation is a healthy practice and, if so, how much seaweed is a proper amount [30, 31]. Moreover, antioxidants from marine products have shown anticancer effects [32]. For example, fucoxanthin is abundant in seaweed and is considered to be an effective anticancer drug. A recent report suggested that fucoxanthin augments apoptosis and reduces tumor-cell proliferation, migration, and invasion [33].

Mushrooms are a vegetable rich in vitamin D. Negative associations have been found between vitamin D and blood pressure [34], endothelial function [35], and diabetes [36]. Vitamin D also showed a protective effect against breast cancer in both American [37, 38] and Japanese women [39].

The negative association between fruit consumption and all-cause mortality has been reported previously [40] and our study supports this association. Furthermore, one study [37] showed that participants who consumed fruits more than 4 days per week had a 34%, 17%, and 42% lower risk for CVD mortality, cancer mortality, and chronic obstructive pulmonary disease, respectively. The consumption of fruit has also been negatively associated with mortality related to digestive tract cancer and esophageal cancer [40].

### Dairy intake and mortality

We found a negative association between intake of dairy products and all-cause mortality, which is consistent with other studies [23]. The Japan Collaborative Cohort [23] reported that a dairy-rich dietary pattern was associated with a 35% and 30% reduction of all-cause mortality for men and women, respectively. The protective effect of dairy products on all-cause mortality may have its basis in a reduction of cardiovascular risk factors, such as hypertension, diabetes, and dyslipidemia. Systematic review and meta-analysis studies have reported that dairy products have a protective effect against type 2 diabetes [41] and hypertension [42]. Furthermore, a randomized dietary intervention study revealed that three daily servings of dairy products led to a significant reduction in mean daytime ambulatory systolic blood pressure [43]. Moreover, a meta-analysis of prospective cohort studies [44] concluded that increased consumption of dairy foods may be associated with a reduced risk of breast cancer. The results of the European Prospective Investigation into Cancer and Nutrition suggested evidence for a possible protective role of dairy products against colorectal cancer risk [45]. These studies support the conclusion that dairy products have a protective effect against cancer as well as total mortality risk.

However, a Mendelian randomization study (MR) [46] in a Danish population reported that there was not strong evidence of an observational or genetic association between milk intake and all-cause or cause-specific mortality. Moreover, a systematic review and meta-analysis of observational cohort studies of mostly Caucasians [47] also found no evidence for a decreased or increased risk of all-cause mortality associated with milk consumption by adults. Therefore, the association between milk consumption and all-cause death risk remains controversial and requires more prospective and/or MR studies to elucidate and to consider potential confounders, such as ethnic variation.

### Fish intake and mortality

Sea fish is a good source of omega-3 polyunsaturated fatty acids. Fish oil, high in EPA (eicosapentaenoic acid) and DHA (docosahexaenoic acid), has been reported to have a protective effect against CVD [48] and many inflammatory diseases. Moreover, a clinical trial also revealed that dietary approaches to control hypertension are effective for reducing blood pressure when low-fat protein-rich foods and fish are substituted for red meats as sources of protein in the diet [49].

### Tea intake and mortality

The health benefits of coffee and tea have been studied [50, 51]. A meta-analysis with prospective cohorts [50] reported that consumption of one cup of green tea per day was associated with a 5% lower risk of CVD mortality and a 4% lower risk of all-cause mortality. Moreover, green tea consumption was significantly inversely associated with CVD and all-cause mortality, whereas black tea consumption was significantly and inversely associated with all cancer and all-cause mortality.

### Sugar-sweetened beverages/fermented foods/fatty meats intake and mortality

Fatty meats, sweetened beverages and fermented foods were identified to be associated with all-cause mortality in our study. Fatty meats, sweetened beverages and fermented foods feature high-fat, high-sugar, and high-salt properties, respectively, and have been associated with cardio-metabolic disorders [52, 53]. Prospective studies [54*–*57] lend support showing that these foods were associated with mortality, especially cardiovascular mortality and specific cancers.

### Study strengths and weaknesses

Our study has several strengths. First, the study population was a national representative sample, such that the results could be extended to the entire population of Taiwan and other ethnic Chinese populations with similar dietary cultures. Second, this study demonstrated the use of a food score, the Taiwanese Eating Approach score, with beneficial effects on all-cause and cardiovascular, cancer (elder adults) and other-causes mortality, among a young adult population that has been followed for 18 years.

The study has some limitations. First, our study population was composed of ethnic Chinese, and therefore caution should be used when applying the results to other ethnic populations. Moreover, the association between the food score and mortality may vary due to the distribution of the food score among differential populations. However, the significantly negative association between healthy foods and mortality has been demonstrated in similarly designed studies of other populations. Second, some residual confounding effects may not have been sufficiently controlled because we did not collect all the information (unmeasured potential confounders, such as sleep and actual socioeconomic status) that could confound the association between diet and mortality. Third, food intake and frequency were measured only once at baseline and without data on the amounts of food intake. Therefore, the distribution of intake frequency may have changed with time which may have attenuated the dietary effects to some extent and as such inevitable potential misclassification may exist. The measurement error may also cause non-differentiable misclassification and have slightly affected the results, because the exposure was measured before the outcome ascertainment. Fourth, we did not have information on the intensity of physical activity. Therefore, the classification of regular physical activity may not be replicable. Fifth, the association between TEA score and mortality was conducted among an elderly population as a sensitivity analysis and the competing risk was not adjusted in the elderly population. The association in this elderly population may be slightly biased. Sixth, since this study was an observational design, the association does not imply a causal relationship.

## Conclusion

A dietary pattern which we discovered and named as the TEA (Taiwanese Eating Approach) diet consists of twelve predictive food items or food groups. The more vegetables (including seaweed and mushrooms), fruit, tea, milk, fish (deep sea or fresh water ones) and other seafoods were consumed and the less fatty red meats, fermented vegetable, and sweetened beverages were ingested, the lower the mortality related to cardiovascular, other causes, and all-causes was observed.

## Supporting information

S1 Fig Flow chart of study sample. (PPTX)

S1 File (DOCX)
