# Supplementary material for: The Healthy Taiwanese Eating Approach is inversely associated with all-cause and cause-specific mortality: A prospective study on the Nutrition and Health Survey in Taiwan, 1993-1996
Source: PLoS One. 2021 May 6;16(5):e0251189. doi: 10.1371/journal.pone.0251189 (PMC8101962; doi:10.1371/journal.pone.0251189)
Supplement: S1 Fig — (PPTX) [file pone.0251189.s001.pptx]

## Slide 1
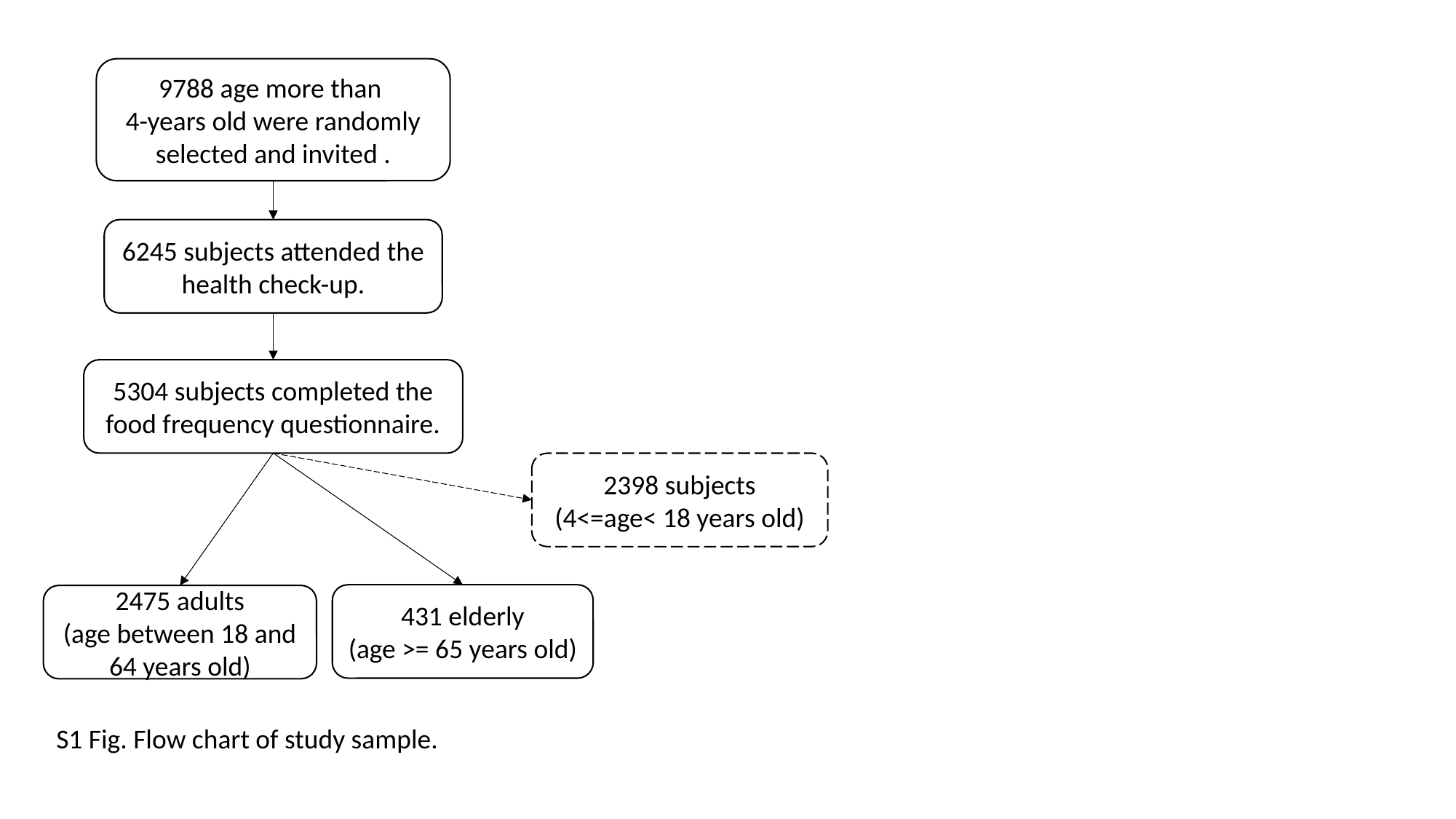

9788 age more than
4-years old were randomly selected and invited .
6245 subjects attended the health check-up.
5304 subjects completed the food frequency questionnaire.
2398 subjects
(4<=age< 18 years old)
431 elderly
(age >= 65 years old)
2475 adults
(age between 18 and 64 years old)
S1 Fig. Flow chart of study sample.
