# Supplementary material for: The Healthy Taiwanese Eating Approach is inversely associated with all-cause and cause-specific mortality: A prospective study on the Nutrition and Health Survey in Taiwan, 1993-1996
Source: PLoS One. 2021 May 6;16(5):e0251189. doi: 10.1371/journal.pone.0251189 (PMC8101962; doi:10.1371/journal.pone.0251189)
Supplement: S1 File — (DOCX) [file pone.0251189.s002.docx]

**The Healthy Taiwanese Eating Approach is inversely associated with all-cause and cause-specific mortality: A prospective study on the Nutrition and Health Survey in Taiwan, 1993-1996**

Shao-Yuan. Chuang^1^, Hsing-Yi Chang^1,3^, Hsin-Ling Fang^1^, Shu-Chen Lee^2^, Yueh-Ying Hsu^1^, Wen-Ting. Yeh^1^, Wen-Ling. Liu^1^, Wen-Harn. Pan^1,2^

^1^Institute of Population Health Science, National Health Research Institutes, Miaoli, Taiwan, R.O.C.

^2^Institute of Biomedical Sciences, Academic Sinica, Taipei, Taiwan, R.O.C.

^3^Institute of Public Health, National Yang Ming University, Taipei, Taiwan, R.O.C.

The author(s) declare no competing interests.

Running title: Diet, and mortality

Key words: Food, cardiovascular mortality, ethnic Chinese

Words text: 2883

Tables and figures: 3 tables and 1 figures

Corresponding author: Shao-Yuan Chuang &Wen-Harn Pan

E-mail: [chuangsy@nhri.org.tw](mailto:chuangsy@nhri.org.tw)

Address:No.35.,Keyan Road, Miaoli County 35053, Taiwan, R.O.C.

Phone: +886-37-364137

Fax: +886-37-586261

Corresponding author: Wen-Harn Pan

E-mail: [pan@ibms.sinica.edu.tw](mailto:pan@ibms.sinica.edu.tw)

Address: Institute of Biomedical Science, Academia Sinica, 128 Sec. 2, Academia

Road, Nankang, Taipei 115 Taiwan, ROC

Phone: +886-2-2789-9121

Fax: +886-2-2789-3047

S1 Table. The association between Taiwanese Eating Approach (TEA) score and mortality.

|  | All-cause mortality | Cardiovascular mortality | Cancer mortality | Other-cause mortality |
| --- | --- | --- | --- | --- |
| Death events, n | 288 | 100 | 56 | 132 |
| Mortality rate* | 56.6 | 19.7 | 11.0 | 25.9 |
|  |  |  |  |  |
| Hazard ratio** | 0.84 | 1.05 | 0.58 | 0.85 |
| (95% confidence intervals) | (0.75~0.94) | (0.85~1.28) | (0.44~0.75) | (0.72~1.00) |
| p-value | 0.0026 | 0.71 | <0.001 | 0.05 |

*: per 1000 person-years

**: per score of Taiwanese Easting Approach in the multivariable model with adjusted for age, sex, exercise, smoking, drinking, education, obesity, number of self-reported diseases, systolic BP, diastolic BP, triglycerides, HDL-cholesterol, and LDL-cholesterol, sugar intake (times/week), belt-nuts (times/week)

S2 Table. Missing proportion in food frequency questionnaire

| Age, yes | 43.49 +/- 13.4 |  |
| --- | --- | --- |
| Sex, male | 46.55% |  |
| Body mass index, kg/m^2^ | 23.85+/-3.85 |  |
| **Education** |  |  |
| 0: Informal school | 54 (2.19%) |  |
| 1 No schooling, no literacy | 227 (9.18%) |  |
| 2 No school, literacy | 22 (0.89%) |  |
| 3 Elementary | 923 (37.31%) |  |
| 4 Junior high school | 362 (14.63%) |  |
| 5 Senior High School | 621 (25.10%) |  |
| 6 Undergraduate | 253 (10.23%) |  |
| 7 Graduate | 10 (0.40%) |  |
|  |  |  |
|  |  |  |
| Variables | Original | Imputation |
| **Smoking** |  |  |
| NONE-SMOKING | 1529 (61.78%) | 1656 (66.91%) |
| CURRENT-SMOKIN | 601 (24.28%) | 659 (26.63%) |
| Former | 146 (5.90%) | 160 (6.46%) |
| Missing (reject to answer) | 199 (8.04%) |  |
|  |  |  |
| **Drinking** |  |  |
| none-drinking habits | 1011 (40.85%) | 1069 (43.18%) |
| Former | 124 (5.01%) | 126 (5.09%) |
| Current DRINKING | 1281 (51.76%) | 1280 (51.73%) |
| Missing (reject to answer) | 59 (2.38%) |  |
